# Prospective clinical trial of 12-fraction carbon-ion radiotherapy for primary renal cell carcinoma

**DOI:** 10.18632/oncotarget.26539

**Published:** 2019-01-01

**Authors:** Goro Kasuya, Hiroshi Tsuji, Takuma Nomiya, Hirokazu Makishima, Yasuo Haruyama, Gen Kobashi, Kazuhiko Hayashi, Daniel K. Ebner, Tokuhiko Omatsu, Riwa Kishimoto, Shigeo Yasuda, Tatsuo Igarashi, Mototsugu Oya, Koichiro Akakura, Hiroyoshi Suzuki, Tomohiko Ichikawa, Jun Shimazaki, Tadashi Kamada, the Working Group for Genitourinary Tumors

**Affiliations:** ^1^ Hospital of the National Institute of Radiological Sciences, National Institutes for Quantum and Radiological Science and Technology, Chiba, Japan; ^2^ Department of Radiology, Joban Hospital, Iwaki, Japan; ^3^ Department of Public Health, Dokkyo Medical University, Tochigi, Japan; ^4^ Osaka Heavy Ion Therapy Center, Osaka, Japan; ^5^ Harvard TH Chan School of Public Health, Boston, MA, USA; ^6^ Department of Radiation Oncology, Chiba Rosai Hospital, Chiba, Japan; ^7^ Department of Urology, Seirei Sakura Citizen Hospital, Chiba, Japan; ^8^ Center for Frontier Medical Engineering, Chiba University, Chiba, Japan; ^9^ Department of Urology, Keio University School of Medicine, Tokyo, Japan; ^10^ Department of Urology, Japan Community Health Care Organization Tokyo, Shinjuku Medical Center, Tokyo, Japan; ^11^ Department of Urology, Toho University Sakura Medical Center, Chiba, Japan; ^12^ Department of Urology, Graduate School of Medicine, Chiba University, Chiba, Japan

**Keywords:** carbon-ion radiotherapy, renal cell carcinoma, prospective study, radiation therapy, renal function

## Abstract

The aims of this study were to clarify the safety and efficacy of 12-fraction carbon-ion radiotherapy (CIRT) for primary renal cell carcinoma (RCC) and to confirm the recommended dose in a prospective clinical trial.

This clinical trial was planned as a non-randomized, open-label, single-center phase I/II study of CIRT monotherapy. The incidence of acute adverse events was the primary endpoint. Dose-limiting toxicities (DLTs) were defined as grade ≥3 skin, gastrointestinal tract, or urologic adverse events.

Based on the eligibility criteria, 8 patients with primary RCC, including 3 medically inoperable patients and 5 patients with tumors >4 cm, were enrolled. Of the 8 patients, 5 were treated with 66 Gy (relative biological effectiveness [RBE]), and subsequently, the dose was escalated to 72 Gy (RBE) for the remaining 3 patients. The median follow-up time was 43.1 months. No DLTs were observed at any dose level though the end of follow-up. Although 1 patient died of pneumonia 3 months after CIRT, which was determined to be unrelated to CIRT, no grade 3 or higher adverse events were observed, and both local control and cancer-specific survival rates were 100%.

In conclusion, the safety and efficacy of CIRT hypofractionation using 12-fractions for the treatment of eligible RCC patients, including those with inoperable or tumor size >4 cm, were confirmed in this prospective trial, and a recommended dose of 72 Gy (RBE) was established.

## INTRODUCTION

The gold standard treatment for patients with renal cell carcinoma (RCC) is surgical removal of the primary tumor via partial nephrectomy or radical nephrectomy [1]. In patients with localized small RCCs (<4 cm, stage Ia), ablative therapies such as cryotherapy and radiofrequency ablation are now offered as radical treatments with active surveillance employed for elderly patients or patients with comorbidities [1]. However, no standard radical treatment options are available for RCC patients with large localized tumors (>4 cm) or for those who are ineligible for surgery due to advanced disease stage, comorbidities, advanced age, or refusal of surgery.

The Hospital of the National Institute of Radiological Sciences (NIRS) started using carbon-ion radiotherapy (CIRT) to treat primary RCC in 1997. CIRT offers not only higher dose concentrations but also increased biological efficacy due to an inherently high-linear energy transfer, compared to those of novel x-ray technologies such as stereotactic ablative radiotherapy (SABR)/stereotactic body radiotherapy (SBRT) [2, 3].

We previously reported the results of a pilot study of CIRT for primary RCC [4]. In addition, patients undergoing primary 16-fraction CIRT with long-term follow-up were retrospectively analyzed, with a local control rate of 94.1% and limited severe adverse events in patients without definitive renal comorbidities, such as diabetic nephropathy, sclerotic kidney, or solitary kidney [5]. Subsequently, a prospective clinical trial of 12-fraction CIRT for primary RCC was performed to reduce the patient burden of treatment, aiming to confirm the safety of treatment and dose recommendations. These results are reported herein.

## RESULTS

Five patients were treated with 66 Gy (relative biological effectiveness [RBE]) without dose-limiting toxicity, and subsequently the dose was escalated to 72 Gy (RBE) for the next three patients. No further dose escalation was performed above 72 Gy (RBE). This clinical trial was censored to March 2017 due to difficulty with patient accrual, although the registration time was extended for 2 additional years. The final patient population consisted of these 8 patients, whose characteristics are provided in Table 1. The average age of the 8 patients was 71.0 ± 10.4 years, and the median tumor size was 4.3 (range, 2.9–8.2) cm. Of the 8 patients, 3 were deemed medically inoperable (#2, #6, and #7). One (#1) had an atrophic contralateral kidney, which generally necessitates postoperative dialysis. Among the 8 patients, two histologically- and six radiologically-proven RCCs were evaluated post-CIRT over a median follow-up of 43.1 (range, 3.0‒62.0) months.

**Table 1:** Patient characteristics

Pt. #	Age	Sex	PS	Operability	Reason for no surgery	T stage^❇^	Diagnosis	Dose (Gy [RBE])	Follo-up time (mo)	Tumor Size (cm)		eGFR, mL/min./1.73 m^2^ (grade^*^)			CIRT-related adverse events other than renal (grade^*^)	
										Pre-CIRT	Follow-up end	Pre-CIRT	3 mo post- CIRT	Follow-up end	Acute	Late
1	58	F	0	Yes	Refusal^(a)^	T1b	Biopsy^(d)^	66	62	4.5	3.0	77 (0)	69 (0)	47 (2)	Dermatitis (1)	Dermatitis (1)
2	89	M	1	No	Advanced age	T1b	Imaging	66	3	4.8	4.8	45 (2)	42 (2)^§^		None	-
3	72	M	0	Yes	Refusal^(b)^	T1a	Imaging	66	54	3.7	3.0	69 (0)	64 (0)	56 (2)	Dermatitis (1)	None
4	75	M	0	Yes	Refusal^(b)^	T1b	Biopsy^(d)^	66	52	4.8	3.8	83 (0)	87 (0)	73 (0)	Dermatitis (1)	Dermatitis (1)
5	65	M	0	Yes	Refusal^(b)^	T1b	Imaging	66	48	4.1	2.7	74 (0)	80 (0)	63 (0)	None	None
6	61	M	0	No	Religious reason^(c)^	T1a	Imaging	72	38	2.9	2.0	66 (0)	60 (0)	47 (2)	Dermatitis (1)	None
7	81	M	1	No	Psychosomatic disorders	T1a	Imaging	72	36	3.7	2.6	42 (2)	45 (2)	42 (2)	Dermatitis (1)	None
8	67	M	0	Yes	Refusal	T3a	Imaging	72	24	8.2	7.6	57 (2)	56 (2)	45 (2)	Dermatitis (1)	Dermatitis (1) Proteinuria (1)

^*^According to the National Cancer Institute’s Common Toxicity Criteria version 4.0.

^❇^All cases were N0M0.

^§^Patient #2 died of pneumonia 3 months post-CIRT, which was judged to be a CIRT-non-related death.

^(a)^Due to avoidance of post-surgical dialysis because of atrophic contralateral kidney.

^(b)^#3, #4, and #5 had a past medical history of asthma, angina pectoris, and high risk of cerebral infarction due to cervical artery stenosis, respectively, which were considered high-risk comorbidities for surgery.

^(c)^Refusal of blood transfusion.

^(d)^Clear cell carcinoma.

Abbreviations: Pt, patient; M, male; F, female; PS, performance status; RBE, relative biological effectiveness; f/u, follow-up; mo, month; eGFR, estimated glomerular filtration rate; CIRT, carbon-ion radiotherapy.

No acute worsening of renal function grade or other CIRT-related acute toxicities, including acute systemic symptoms such as nausea or vomiting, were observed, with the exception of grade 1 skin reaction. Late grade ≥2 CIRT-related adverse events external to the kidney were not observed. With respect to renal adverse events, the average decrease in estimated glomerular filtration rate (eGFR) by the end of follow-up was 10.8 mL/min./1.73 m^2^ in 6 patients; the patient with atrophic contralateral kidney (#1) and a patient who died of pneumonia 3 months post-CIRT (#2) were excluded from this analysis. No patients progressed to grade ≥3 chronic kidney disease. No dose-limiting toxicities (DLTs) were observed at any dose level. Based on these results, the recommended dose was determined to be 72 Gy (RBE) and was employed as the higher prescribed dose in this study.

In one patient (#2, an 89-year old male) who died of pneumonia, CIRT was performed on RCC located in the lower pole of his right kidney, with no lung irradiation. He returned to the hospital 3 months later with dyspnea, which on CT was thought to be due to infectious pneumonia. His condition deteriorated after transfer to a local emergency center, and he died the following day. This was judged to be unrelated to CIRT.

**Figure 1:**
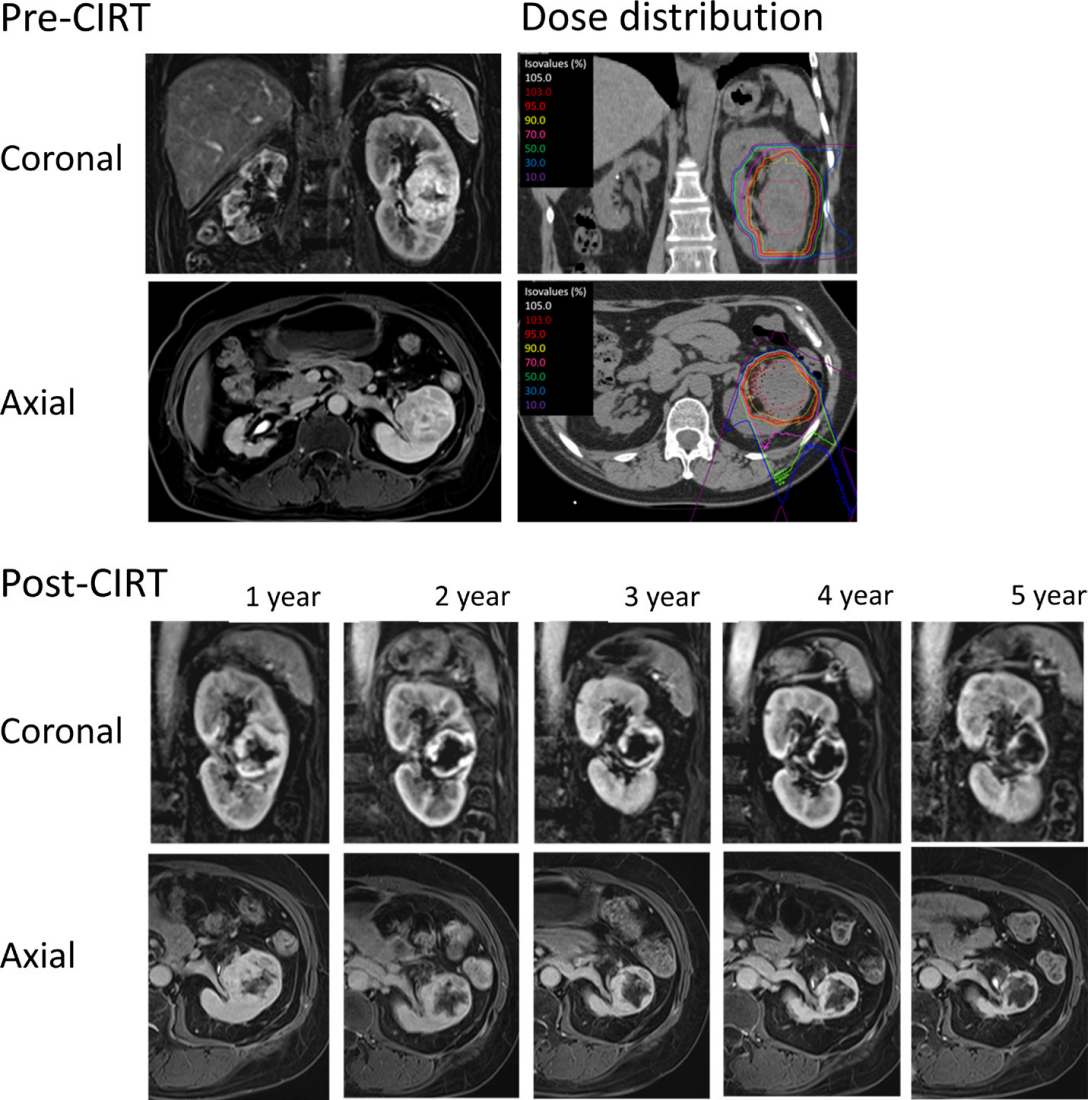
Morphologic changes of the tumor in the left kidney on contrast-enhanced axial and coronal MRI images in patient #1 at pre-, and post-CIRT at 1 to 5 years, as well as dose distribution

None of the 7 living patients received alternative treatment pre- or post-CIRT for RCC, including systemic therapy such as interferon-α or molecular targeted therapy during this study, and both the local control and cancer-specific survival rates were 100%. Figure 1 shows the morphologic changes of the RCC in the left kidney on contrast-enhanced magnetic resonance imaging (MRI) in patient #1 at pre- and post-CIRT at 1 to 5 years, along with the dose distribution.

## DISCUSSION

The only previous studies conducted with CIRT for RCC have been retrospective, primarily 16-fraction CIRT studies [4, 5]. To the best of our knowledge, this is the first prospective trial of primary RCC treated with 12-fraction CIRT, although this phase I/II study was not completed, partly due to insufficient enrollment of patients. The present study achieved low toxicity both within and outside the kidney, with favorable treatment efficacy at a median follow-up of 43.1 months, and established a recommended dose of 72 Gy (RBE).

Following CIRT, the average decrease in eGFR of 6 evaluable patients was 10.8 mL/min/1.73 m^2^. We previously showed an average eGFR decrease of 6.1 mL/min/1.73 m^2^ with a median follow-up of ≥6 years for patients without definitive renal comorbidities such as diabetic nephropathy, sclerotic kidney, or solitary kidney [5]. This prospective study similarly showed an eGFR decrease following CIRT comparable to that after partial nephrectomy or SBRT/SABR [6–8]. In addition, although contralateral kidney atrophy (#1) normally necessitates post-treatment dialysis, patient #1’s eGFR has been maintained for ≥5 years, without grade ≥3 deterioration (Table 1). Limited renal adverse events after CIRT may be due to the highly conformal nature of the dose distribution, which delivers a high-efficacy dose to tumor tissue while sparing the non-tumorous adjacent kidney. These unique characteristics of CIRT may also prevent non-renal adverse events, such as those that occur in the gastrointestinal tract, and acute systemic symptoms, such as vomiting and fatigue, which have been reported after SBRT/SABR [7–9].

Marked shrinkage of tumors was not observed after CIRT (Table 1); however, the necrotic areas in the tumors gradually enlarged with time (Figure 1). The local control rate and cancer-specific survival rates were 100% among the registered patients. This included 3 medically inoperable patients and 5 patients with tumors >4 cm. Excluding 1 patient who died of pneumonia 3 months following treatment, which was judged being unrelated to CIRT, all remained alive with no recurrence without additional therapy for RCC at the end of follow-up (median, 43.1 months). These favorable results are comparable to those reported for standard radical treatments such as nephrectomy, cryoablation, and radiofrequency ablation [10, 11]; furthermore, this prospective study showed equivalent safety and efficacy compared to 16-fraction CIRT.

Based on the absence of grade ≥3 adverse events, the recommended dose for 12-fraction CIRT is 72 Gy (RBE), the higher dose employed herein, which resulted in good treatment outcomes in 3 patients. A phase II trial is needed to confirm the efficacy of this dose. Of note, the biologically effective dose of 72 Gy (RBE) in 12-fraction CIRT in this study is equivalent to that of 80 Gy (RBE) in 16-fraction CIRT when an α/β ratio of 3 or 5 is applied; this dose has previously shown a good treatment effect [5]. This trial is part of an institutional series of trials on hypofractionation, and a succeeding 4-fraction phase I/II clinical trial of CIRT for RCC began in October 2017 at our institution.

This study has some limitations. First, diagnoses based on imaging were made without performing biopsy in 6 of the 8 patients in this study. Although these patients were diagnosed by multiple diagnostic radiologists, discrepancies between imaging and pathological findings can occur [12]. Second, too few patients were accrued, so larger trials are required. Third, this study was conducted at a single institution with limited follow-up; a multi-institutional prospective study involving more patients and long-term follow-up is required.

In conclusion, the safety and efficacy of CIRT hypofractionation using 12 fractions for eligible RCC patients including cases with inoperable or large localized tumors (>4 cm) were confirmed in this prospective trial, and a recommended dose was established.

## MATERIALS AND METHODS

### Protocol and eligibility criteria

Protocol 1203 was initiated by the Protocol Design Committee of the Network Advisory Board for Heavy Ion Therapy at the National Institute of Radiological Sciences. This study was started in April 2013, and the planned registration time was 2 years. The planned number of patients for this study was 10. The planned number of patients at each dose level was 5, and at least two dose levels were planned.

Inclusion criteria for study entry were: 1) biopsy-proven RCC or a definitive diagnosis of RCC by dynamic contrast-enhanced CT and MRI studies; 2) clear cell, chromophobe, or papillary RCC; 3) untreated T1/T2N0M0 or T3/T4N0M0 disease without tumor emboli according to the TNM Classification of Malignant Tumors (7th edition), or N1/M1 disease with an expected favorable prognosis [13]; 4) an Eastern Cooperative Oncology Group performance status of 0–1; 5) age >20 years; and 6) ability to understand and sign an informed consent form. The exclusion criteria were: 1) previous treatment of the target tumor by radiation therapy, 2) prior use of molecularly targeted therapy, 3) requirement for delivery of ≥60% of the prescribed dose to the gastrointestinal tract, 4) maximum tumor size >15 cm, 5) presence of active secondary cancers other than RCC, 6) estimated life expectancy <6 months, 7) current dependence on dialysis, and 8) presence of a serious medical or psychological condition precluding safe administration of treatment.

The trial was conducted in accordance with the ethical standards set forth by the Declaration of Helsinki [14], and all patients satisfying the enrollment criteria were approved by the ethics committee.

### Study design

Protocol 1203 was a non-randomized, open-label, single-center phase I/II study of CIRT monotherapy, designed to establish 12-fraction treatment for RCC. The incidence of acute adverse events was the primary endpoint, and the incidence of late adverse events, local control rate, and survival rate were secondary endpoints. The phase I component of protocol 1203 was a dose-escalation study consisting of 12 fractions administered over 3 weeks. The starting dose in this clinical trial was 66 Gy (RBE), because the biologically effective dose in 16 fractions is close to 72 Gy (RBE) (when α/β was applied as 3 or 5), which was used most frequently in our previous experience [4, 5]. DLTs were defined as grade ≥3 skin, gastrointestinal tract, or urologic adverse events according to the National Cancer Institute’s Common Toxicity Criteria, version 4.0 [15], and the timing of DLT evaluation was 2 years after completion of treatment. The dose per fraction was planned to escalate by 10% following successful initial treatment, as determined by discussion with the protocol committee of the Working Group for Genitourinary Tumors. The planned follow-up observation period for the evaluation of late adverse events, local control, and survival was set ≥2 years after the last patient was registered.

### Treatment

Irradiation fields were established using a three-dimensional planning system based on 2.5‒5-mm-thick CT images. The gross tumor volume was defined as the macroscopic tumor, and the clinical tumor volume was defined as the gross tumor volume +5 mm to account for microscopic invasion. The planning target volume was defined as the clinical tumor volume +10 mm in the cranial and caudal directions and +5 mm in the other dimensions, including the internal and set up margins. For accurate reproduction of the target position, an immobilization device was constructed from a low-temperature thermoplastic sheet. During treatment, patient‒machine alignment was confirmed by overlapping the on-board image taken in a true lateral position using kV X-rays, and the reconstructed two-dimensional image was taken during planning CT, automatically minimizing deviations in skeletal anatomy and the inserted fiducial markers between the two images. Respiratory gating at the end of the expiratory phase was used for CT planning, with positional verification used during treatment [16].

### Follow-up

Each patient was generally examined by blood tests, ultrasonography, and dynamic contrast-enhanced CT and MRI, according to the European Society of Urogenital Radiology guidelines [17], at least once every 3 months for the first 6 months, and then usually every 6 months thereafter.

To evaluate kidney toxicity, eGFR was calculated in all cases according to the formula reported by Matsuo et al. [18]. Other adverse events including kidney function were evaluated according to the National Cancer Institute’s Common Toxicity Criteria, version 4.0 [15]; the details of chronic kidney function grade were described in our previous report [5]. Acute and late adverse events were evaluated within the first 3 months and ≥3 months after CIRT, respectively.

### Evaluation

Evaluation of eGFR was conducted from pre-CIRT to the end of follow-up. Local failure was defined as either progressive disease according to the modified Response Evaluation Criteria in Solid Tumors [19] or the new appearance of lesions within the target volume. Local control was defined as the absence of detectable local failure. Distant failure was defined as the development of metastatic lesions outside of the kidney. The time to failure was defined as the interval from the start of CIRT to the date of diagnosed recurrence. The survival time was defined as the interval from the start of CIRT to the date of death or last follow-up. The cutoff date for analysis was October 2018.

### Statistics

Both local control and overall survival rates were calculated by the Kaplan‒Meier method using SPSS software (version 20.0; IBM Japan, Ltd., Tokyo, Japan).

## Abbreviations

CIRT: carbon-ion radiotherapy
CT: computed tomography
DLT: dose-limiting toxicity
eGFR: estimated glomerular filtration rate
MRI: magnetic resonance imaging
NIRS: National Institute of Radiological Sciences
RBE: relative biological effectiveness
RCC: renal cell carcinoma
SABR: stereotactic ablative radiotherapy
SBRT: stereotactic body radiotherapy
